# Potent Antitumor Effects of a Combination of Three Nutraceutical Compounds

**DOI:** 10.1038/s41598-018-29683-1

**Published:** 2018-08-15

**Authors:** Vikalp Vishwakarma, Jacob New, Dhruv Kumar, Vusala Snyder, Levi Arnold, Emily Nissen, Qingting Hu, Nikki Cheng, David Miller, Ahia Rael Thomas, Yelizaveta Shnayder, Kiran Kakarala, Terance Ted Tsue, Douglas A. Girod, Sufi Mary Thomas

**Affiliations:** 1 Department of Otolaryngology, University of Kansas Medical Center, 3901 Rainbow Blvd, Kansas City, KS 66160 USA; 2 Department of Anatomy and Cell Biology, University of Kansas Medical Center, 3901 Rainbow Blvd, Kansas City, KS 66160 USA; 3 Department of Pathology, University of Kansas Medical Center, 3901 Rainbow Blvd, Kansas City, KS 66160 USA; 4 Department of Mechanical Engineering Technology, Pittsburg State University, Pittsburg, KS 66762 USA; 5 Department of Cancer Biology, University of Kansas Medical Center, 3901 Rainbow Blvd, Kansas City, KS 66160 USA; 6 Present Address: Amity Institute of Molecular Medicine and Stem Cell Research, Amity University, Uttar Pradesh Noida, India

## Abstract

Head and neck squamous cell carcinoma (HNSCC) is associated with low survival, and the current aggressive therapies result in high morbidity. Nutraceuticals are dietary compounds with few side effects. However, limited antitumor efficacy has restricted their application for cancer therapy. Here, we examine combining nutraceuticals, establishing a combination therapy that is more potent than any singular component, and delineate the mechanism of action. Three formulations were tested: GZ17-S (combined plant extracts from *Arum palaestinum*, *Peganum harmala* and *Curcuma longa*); GZ17-05.00 (16 synthetic components of GZ17-S); and GZ17-6.02 (3 synthetic components of GZ17S; curcumin, harmine and isovanillin). We tested the formulations on HNSCC proliferation, migration, invasion, angiogenesis, macrophage viability and infiltration into the tumor and tumor apoptosis. GZ17-6.02, the most effective formulation, significantly reduced *in vitro* assessments of HNSCC progression. When combined with cisplatin, GZ17-6.02 enhanced anti-proliferative effects. Molecular signaling cascades inhibited by GZ17-6.02 include EGFR, ERK1/2, and AKT, and molecular docking analyses demonstrate GZ17-6.02 components bind at distinct binding sites. GZ17-6.02 significantly inhibited growth of HNSCC cell line, patient-derived xenografts, and murine syngeneic tumors *in vivo* (P < 0.001). We demonstrate GZ17-6.02 as a highly effective plant extract combination and pave the way for future clinical application in HNSCC.

## Introduction

Despite advances in therapy, overall survival of head and neck squamous cell carcinoma (HNSCC) has marginally improved over the past 30 years^1^. Treatments are intensive and often result in severe toxicity^2^. One-third to one-half of survivors develop second primary tumors. With these dismal outcomes, there is great need for improved HNSCC therapies.

Nutraceuticals provide a powerful alternative to prevent and treat HNSCC because of their safety and general acceptance. In HNSCC preclinical models, promising antitumor efficacy with isothiocyanate^3^, luteolin^4^, resveratrol^5^, and genistein have been reported^6^. HNSCC nutraceutical clinical trials include: vitamin A derivatives^7^, curcumin^8^, green tea extract^9^, soybean extract^10^, and lycopene^11^. However, these are limited by studying prevention rather than treatment, and have had little efficacy and adoption into practice. As combining anticancer agents has proven to reduce side effects of single agents and potentiate antitumor effects, we sought to investigate if combining nutraceuticals may create an improved effect, and allow for a lower concentration of inhibitor to be used.

*Curcuma longa* is a widely studied nutraceutical^12^. Its active ingredient, curcumin, inhibits nuclear factor-κB (NF-κB), mitogen activated protein kinase (MAPK), vascular endothelial growth factor (VEGF), and epidermal growth factor receptor (EGFR)^13,14^. However, curcumin has poor bioavailability^15^. As such, analogs of curcumin and nanoparticle encapsulation techniques have been designed to increase bioavailability^16,17^. Further, the combination of curcumin with additional nutraceuticals potentiates efficacy^18^, and the combination of curcumin provides additive benefit to chemotherapeutics^19^.

*Arum palaestinum* is widely cultivated in Palestine, and has been used in the treatment of cancer in Palestine for many years^20^. Ethanolic extracts of *Arum palaestinum* have shown antitumor efficacy against breast cancer, leukemia, and prostate cancer^21,22^. Yet, little has been done to characterize *Arum palaestinum’s* mechanism of action.

The alkaloids from the plant *Peganum harmala* are known to contain a wide spectrum of medicinal properties. The main constituent, harmine, is an inhibitor of monoamine oxidase, and also demonstrates anti-tumor effects^23^. Harmine intercalates and damages DNA^24^, and mitigates chemotherapy resistance by interfering with drug efflux^25^. Further, harmine decreased proliferation of various tumor lines, while having little effect on normal cells^26^.

Chemotherapies are often given to patients in combination. The aim of this study was to determine whether a potentiated effect could be achieved by combining nutraceuticals. Given the documented success of combination therapy with curcumin^18,19^, this was used as a starting point and included *Arum palaestinum* and *Peganum harmala* for their proposed anti-cancer activity in HNSCC. We assessed the combined plants, comparing a dried extract of the three plants (GZ17-S), a synthetic version of the extract (GZ17-05.00) and the three major anti-cancer agents found in the original plants (GZ17-6.02). Our results demonstrate a highly effective combination for use in HNSCC, more potent than any component used singularly, when assessed in preclinical models. We delineate the mechanism of action, and provide evidence of a useful biomarker for future clinical study.

## Results

### Combination of curcumin, harmine, and isovanillin demonstrates potent cytotoxicity in cancer cell lines

**Figure 1:**
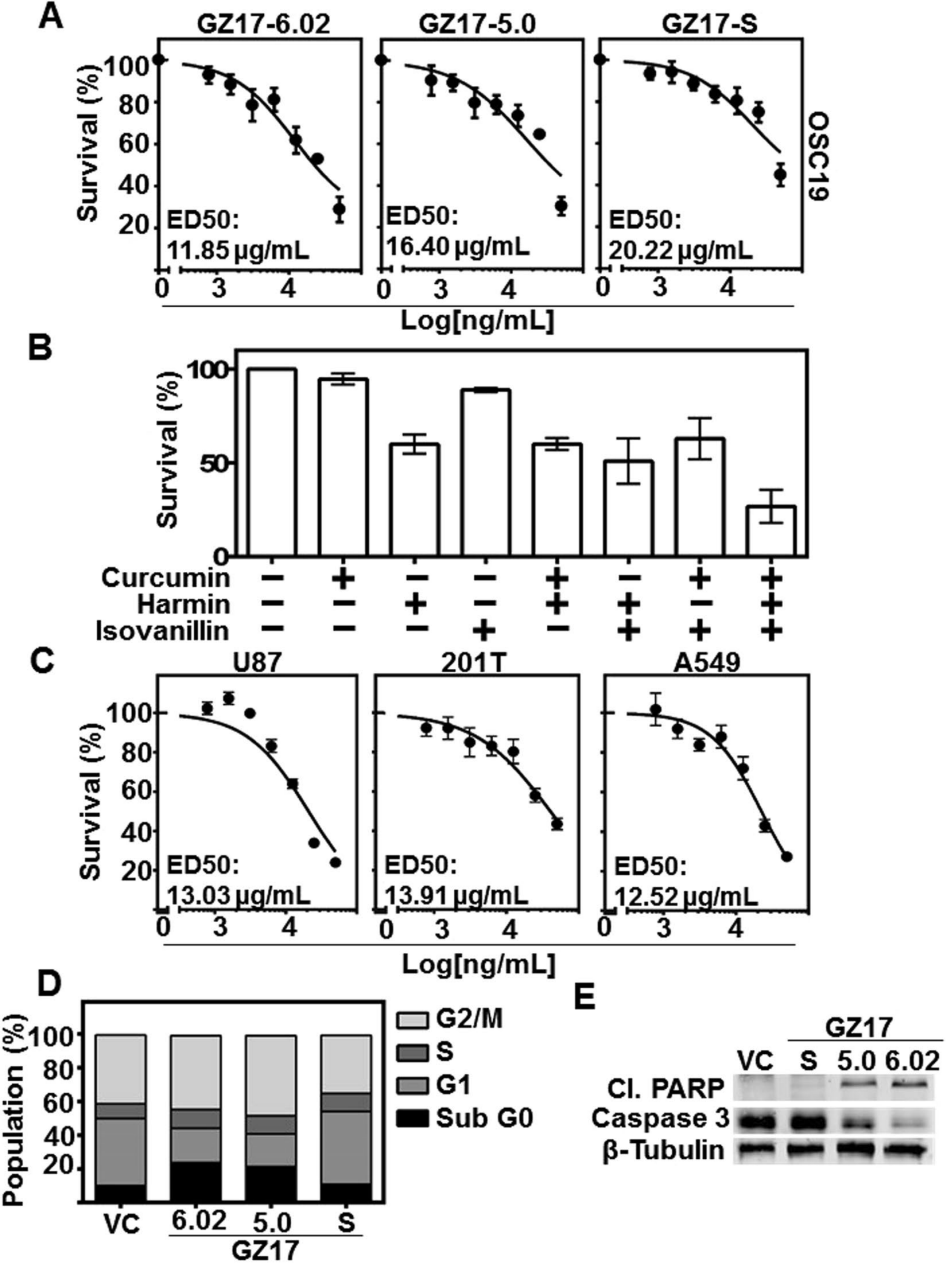
GZ17 formulations are cytotoxic to HNSCC, and have a potentiated effect compared to individual components. (**A**) OSC19 (4 × 10^3^ cells/well in triplicate) were treated with various concentrations of GZ17-6.02, -5.0 and -S. Effective dose 50 (ED_50_) was calculated with non-linear curve fit using GraphPad Prism software. Cumulative data represents three individual experimental repeats and error bars represent ± SEM. (**B**) OSC19 (4 × 10^3^ cells/well in triplicate) were treated with curcumin, harmine or isovanillin or combination of two components, each in a ratio representative of GZ17-6.02 in a final dose of 50 ug/mL for 48 h. (**C**) Glioblastoma (U87), and lung cancer lines (201T and A549) were treated with various concentrations of GZ17-6.02 to determine ED_50_ concentration. (**D**) HNSCC cells (OSC19; 2 × 10^5^ cells) were treated with vehicle control or ED_50_ concentrations of GZ17-6.02, -05.00 or –S for 72 h and analyzed by flow cytometry. The percentage of cells in various cell cycle stages is represented of each treatment of GZ17 formulation at ED_50_ concentration. Graph represents cumulative results from three independent experiments. (**E**) Representative immunoblot of apoptotic markers cleaved-PARP and caspase-3. β-tubulin levels demonstrate equal loading of protein across lanes.

To determine the dose response to the formulations, varying concentrations of GZ17-formulations were tested on HNSCC cell lines (0, 0.78125, 1.5625, 3.125, 6.25, 12.5, 25, 50 ug/mL). GZ17-6.02 demonstrated greatest cytotoxicity (OSC19 cell ED_50_ = 11.85 µg/ml; UM-SCC-1 cell ED_50_ = 13.03 µg/ml; HN5 cell ED_50_ = 13.73 µg/ml) as compared to GZ17-5.00 and GZ17-S (Fig. 1A, and Supplemental Fig. 1A). At 50 ug/mL no formula caused complete cytotoxicity, and therefore ED_50_ concentration was used for further studies. Additionally, poor cytotoxicity was observed in Het1A, an immortalized esophageal line from a cancer free patient (Supplemental Fig. 1B).

To characterize cytotoxic effects of curcumin, harmine, and isovanillin in GZ17-6.02, we treated HNSCC cells with vehicle control (ethanol), single or double combination each in a ratio representative of GZ17-6.02 in a final dose of 50 ug/mL for 48 h. GZ17-6.02 demonstrated improved attenuation of viability, which was superior to single or double combination of agents (Fig. 1B). The cytotoxic effects of the combination are additive in nature (Supplemental Fig. 1C). This is the first report of the additive effects of such a combination, and demonstrates efficacy at lower concentrations than any singular component.

Additionally, to assess this combination across multiple tumor types, we assessed the proliferation of glioblastoma line U87, and lung cancer lines 201 T and A549. GZ17-6.02 was cytotoxic in these cell lines as similarly to the HNSCC lines tested (Fig. 1C). These data demonstrate combination therapy using multiple nutraceutical components decreases cancer cell proliferation.

### GZ17-6.02 and −05.00 induce apoptosis through caspase-3 activation and PARP cleavage

To understand the mechanism underlying GZ17 anti-proliferative effects, we treated HNSCC cells for 72 h at the ED_50_ dose. GZ17-05.00 and GZ17-6.02 induced significant apoptosis (24% and 22%, respectively, P < 0.05) as observed by the sub G0 fraction of cell cycle analysis in treated cells (Fig. 1D). To gain insight into the mechanism of GZ17-induced apoptosis, levels of poly-ADP ribose polymerase (PARP) and caspase-3 were evaluated using immunoblot. Treating cells with GZ17-6.02 and GZ17-05.00 at ED_50_ concentrations increased cleaved PARP and decreased caspase-3 (Fig. 1D), indicators of treatment-induced apoptosis.

### GZ17-6.02 mitigates migration and invasion of HNSCC cells, and endothelial cell tubule formation

**Figure 2:**
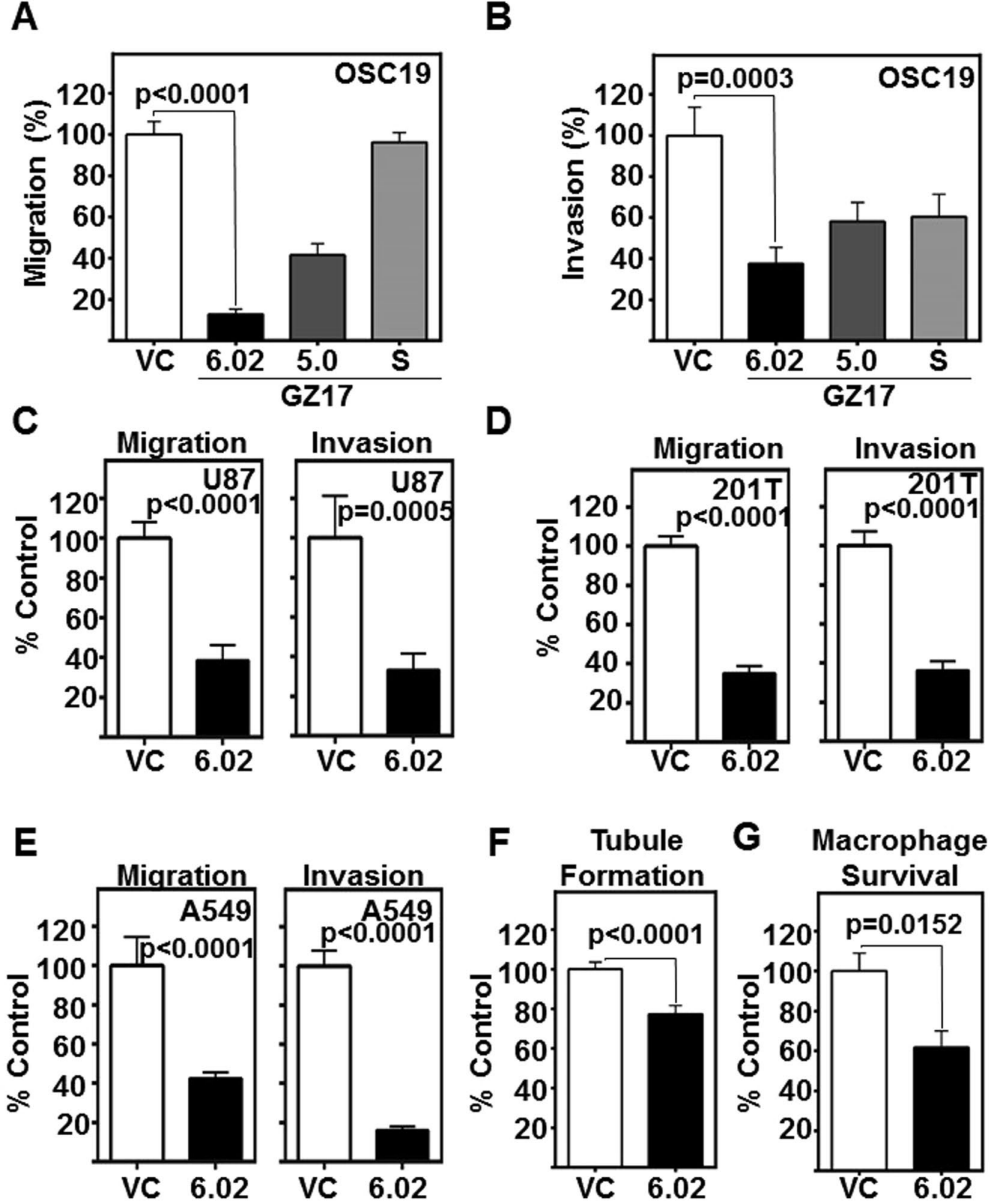
GZ17 formulations mitigate HNSCC invasion and migration, and angiogenesis. (**A**,**B**) HNSCC cells (OSC19; 2 × 10^3^ cells/ well, plated in triplicate) were treated with vehicle control or ED_50_ concentrations of GZ17-6.02, -05.00 or -S. Cell migration and invasion was assessed at 24 h. The number of cells that (A) migrated or (B) invaded were counted and normalized to the cell viability. Percent migration or invasion relative to the vehicle control is depicted in the graphs. Cumulative data represents three individual experimental repeats and error bars represent ± SEM. (**C**,**D**,**E**) Glioblastoma (U87), and (**D** and **E**) lung cancer (201T and A549) were assessed for GZ17-6.02 inhibition of migration and invasion. Cumulative data represents three individual experimental repeats and error bars represent ± SEM. (**F**) GZ17-6.02 attenuates the angiogenic potential of HUVEC *in vitro*. HUVEC cells were treated with the ED_50_ dose (derived from OSC19) of GZ17-6.02 and imaged 6 h after treatment and tubule formation was assessed. Total tube length analyzed using Pipeline software from 15 random fields from each repeat, and normalized to vehicle control treated cells (VC). Cumulative data represents three individual experimental repeats and error bars represent ± SEM. (**G**) GZ17-6.02 inhibits tumor-promoting macrophage survival. Macrophage cell line Thp1 were treated with 60 µg/ml GZ17-6.02 for 48 h. Viable cells were counted using trypan blue dye exclusion. Graph represents cumulative results from five independent experiments and error bars represent ± SEM.

No treatment options are available for the metastatic spread of HNSCC, and patients with metastasis have a 5-year survival rate of less than 20%^27^. We assessed the effects of GZ17-formulations on HNSCC migration and invasion, prerequisites to metastasis. GZ17-6.02 (ED_50_ dose) was most effective in significantly inhibiting migration (p < 0.0001, Fig. 2A, Supplemental Fig. 2A); and invasion (p = 0.0003, Fig. 2B, Supplemental Fig. 2B). Additionally, we assessed the inhibitory effects of GZ17-6.02 against glioblastoma and lung cancer cell line migration and invasion, and observed significant reductions in U87 (Fig. 2C), 201T (Fig. 2D), and A549 (Fig. 2E) migration and invasion.

Angiogenesis plays a critical role in HNSCC progression, however, no antiangiogenic therapies are approved for use in HNSCC^28^. Previously, curcumin has been reported to limit HUVEC tubule formation^29^, and vascular tubule mimicry^30^. We tested the efficacy of GZ17-6.02 (ED_50_ dose) to inhibit endothelial tubule formation, an *in vitro* indicator of angiogenesis^31^. GZ17-6.02 significantly decreased the length of HUVEC endothelial tubules compared to the vehicle control (p = 0.0001, Fig. 2F).

Macrophage infiltration is frequently associated with an inflammatory reaction, and tumor-associated macrophages facilitate tumor progression^32^. We tested the ability of GZ17-6.02 to attenuate macrophage infiltration into tumors using a 3-dimensional cell culture device called the metastatic mimetic device (MMD)^33^. HNSCC cells embedded in a collagen matrix were subjected to macrophage infiltration in the MMD in the presence of vehicle control or GZ17-6.02. GZ17-6.02 effectively reduced macrophage survival (Fig. 2G) and subsequently infiltration through the collagen plugs (Supplemental Fig. 2C). These cumulative data demonstrate that GZ17-6.02 significantly mitigates *in vitro* tumor progression.

### GZ17-formulations modulate key signaling molecules in HNSCC

**Figure 3:**
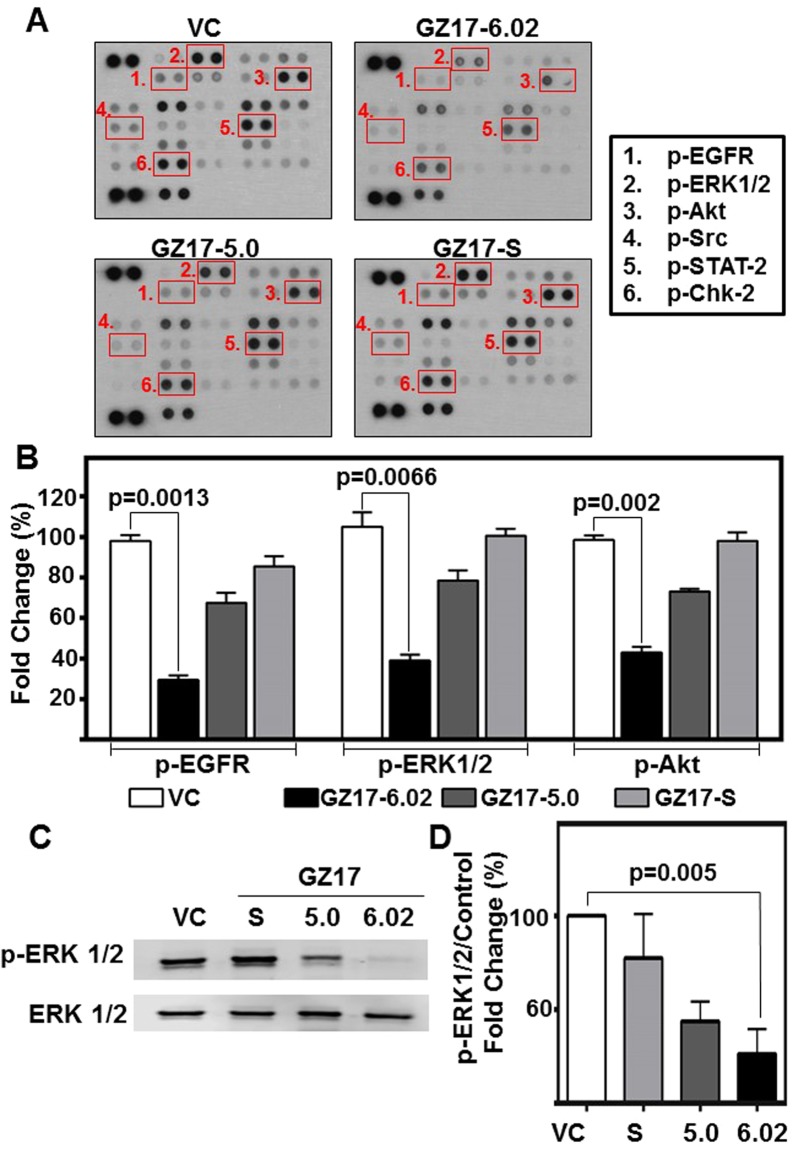
GZ17 formulations modulate levels of several phospho-proteins in HNSCC. (**A**) HNSCC cells (OSC19; 2 × 10^5^ cells) were treated with vehicle control or ED_50_ concentrations of GZ17-6.02, -05.00 or –S for 48 h. Representative dot-blot image from phospho-kinase array. (**B**) Densitometric analyses of dot-blot image signals from GZ17 treated lysates normalized to those from vehicle control and presented as fold change in protein levels. The graph represents cumulative data from two independent experiments. Error bars represent ± SEM. (**C**) HNSCC cells (OSC19; 2 × 10^5^ cells) were treated with ED_50_ concentrations of GZ17-6.02, -05.00, –S or vehicle control for 72 h. Immunoblot was performed for phospho-ERK1/2 and total ERK1/2 as loading control. Image is representative of three independent experimental repeats. (**D**) Densitometric analyses of signals from immunoblots normalized to loading control and presented as fold change in protein levels relative to vehicle control treated cells. Error bars represent ± SEM.

GZ17-formulations demonstrate significant anti-cancer effects *in vitro*. In order to assess the mechanism of action, and to understand the additive effects observed, we used a phospho-protein array. Phosphorylation of several proteins including Src, ERK1/2, EGFR, Akt, STAT-2 and Chk-2 were significantly reduced in GZ17-treated HNSCC (ED_50_ dose) (Fig. 3A,B). These are important mediators of HNSCC proliferation, migration and survival.

ERK1/2 is a regulator of HNSCC proliferation, and increased levels are associated with advanced disease^34^. To validate phospho-protein array results, we evaluated pERK1/2 by immunoblot at both a short time interval when induced by EGF (5 m) and long interval (72 h) (Fig. 3C,D and Supplemental Fig. 2D). These data identify that GZ17-6.02 inhibits phosphorylation of ERK1/2, an important molecular mediator of HNSCC progression.

### GZ17-6.02 components bind with high affinity to EGFR, ERK1/2, and Akt1

**Figure 4:**
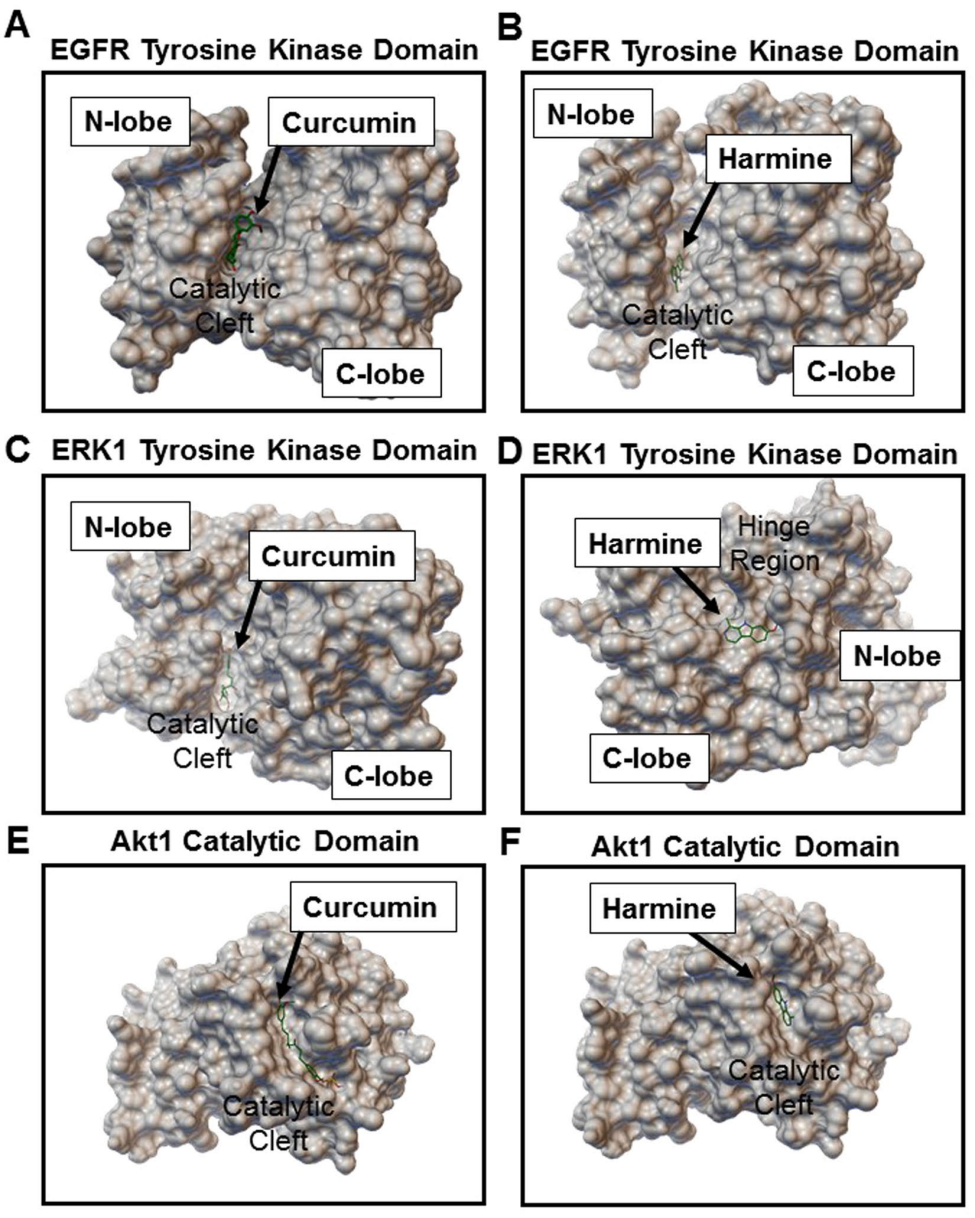
Curcumin and harmine bind with high affinity to distinct sites on EGFR, ERK1 and Akt-1. Binding conformation of top ranked docked poses of (**A**,**C**,**E**) curcumin and (**B**,**D**,**F**) harmine on EGFR tyrosine kinase domain, ERK1 tyrosine kinase domain and Akt1 catalytic domain, respectively. Further details, including detailed labeling of interactive amino acid residues are provided in Supplemental Figs 3–6.

To further understand the mechanism of GZ17-6.02 active components, we carried out molecular docking analyses on the targets identified. Curcumin and harmine, as well as their metabolites, demonstrated high binding affinity to several targets (Supplemental Table 2). Both inhibitors, and their metabolites, demonstrated high affinity towards distinct binding sites on EGFR, ERK1/2, and Akt1 (Fig. 4, and Supplemental Figs 3–6). Curcumin exhibited best binding affinity of −6.6 Kcal/mol to Akt1 and −6.5 Kcal/mol to EGFR. Whereas, harmine showed best binding affinity with −6.3 Kcal/mol to Akt1 and −6.8 Kcal/mol to EGFR. Isovanillin did not have significant binding to the targets assessed. Simultaneous interaction of two inhibitors to the target may explain the improved efficacy of the combination.

### GZ17-6.02 enhances cisplatin efficacy

**Figure 5:**
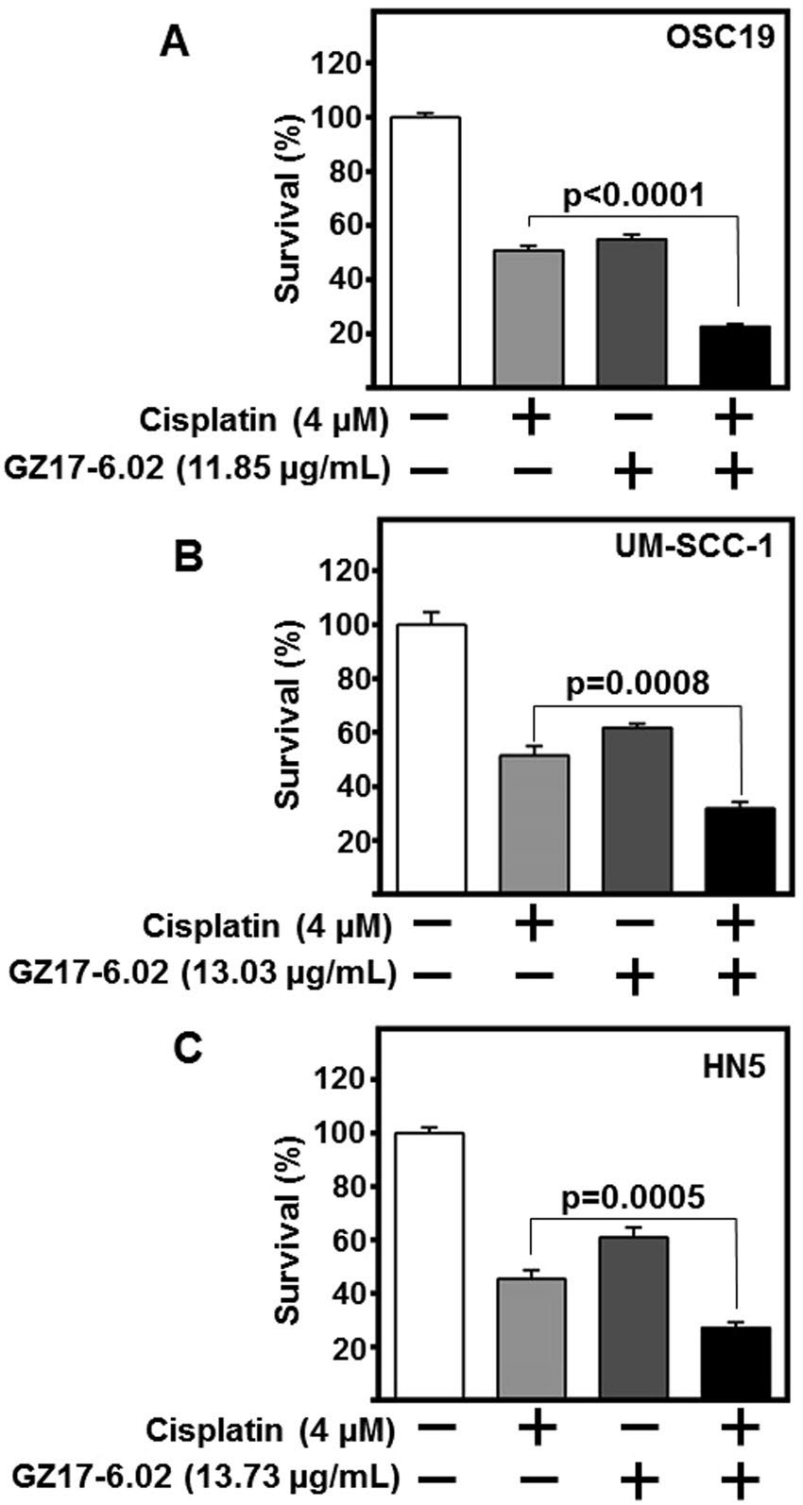
GZ17-6.02 in combination with cisplatin potentiates HNSCC cytotoxicity. HNSCC cells (**A**) OSC19, (**B**) HN5, and (**C**) UM-SCC-1; (2 × 10^3^ cells/well) in triplicate were treated with respective ED_50_ concentrations of GZ17-6.02, cisplatin (4 µM), combination of both GZ17-6.02 and cisplatin, or vehicle control for 72 h. Cell survival was assessed by CyQUANT assay. Obtained values were normalized to vehicle control and depicted as fold change in cell survival. Cumulative data represents three individual experimental repeats plated in triplicate and error bars represent ± SEM.

Platinum-based chemotherapy is a standard choice of treatment for HNSCC^35^. However, despite new protocols, tumor resistance to cisplatin remains a significant hurdle to HNSCC treatment. We assessed the combinatorial effect of GZ17-6.02 (ED_50_) with cisplatin (4.0 µM) in HNSCC cell lines. Combination of GZ17-6.02 and cisplatin significantly reduced HNSCC cell survival as compared to cisplatin or GZ17-6.02 treatments alone (p < 0.0008, Fig. 5A–C). Combination of radiation and GZ17-6.02 found GZ17-6.02 to have greater cytotoxicity than 3, 6, or 9 Gy of radiation alone (Supplemental Fig. 7), with no additive benefit of radiation in combination. This demonstrates GZ17-6.02 treatment potentiates effects of standard HNSCC chemotherapy.

### GZ17-6.02 effectively inhibits HNSCC tumor growth *in vivo*

**Figure 6:**
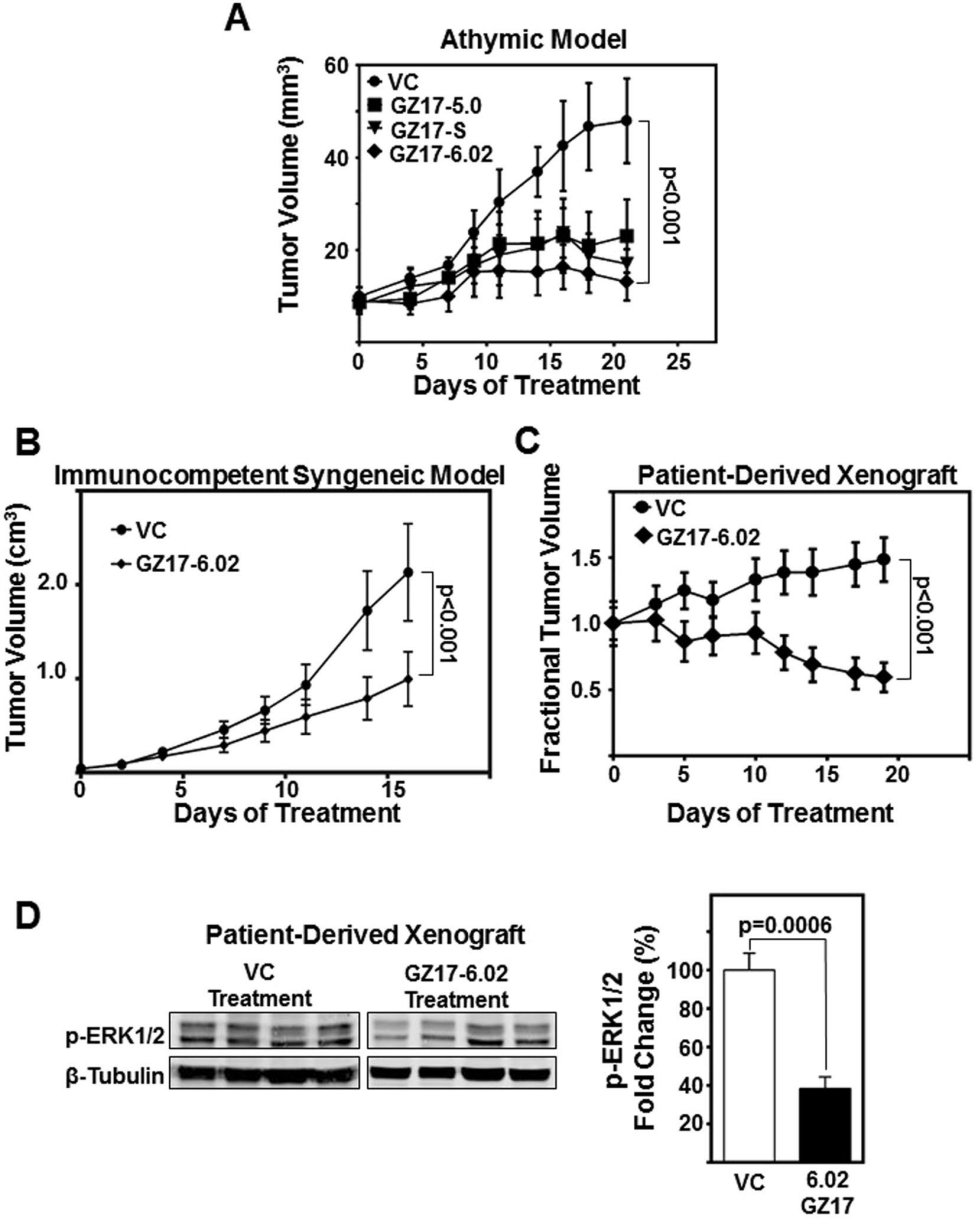
GZ17-6.02 demonstrate significant reduction in HNSCC tumor growth *in vivo*. (**A**) HNSCC cells (OSC19; 1 × 10^6^ cells) were inoculated subcutaneously on the flanks of athymic nude-*Foxn1*^*nu*^ mice. Five mice per group were treated intratumorally with vehicle control (saline), or 15 mg/kg/d of GZ17-06.02, GZ17-5.0 or GZ17-S for three weeks. The graph depicts tumor volumes measured with a vernier caliper over the course of the experiment. Error bars represent ± SEM. (**B**) Immunocompetent SCC/vII tumor bearing C3H mice were treated with GZ17-6.02 (100 mg/kg/day in 1% carboxymethylcellulose (CMC) suspension) or VC (1% CMC) by oral gavage (N = 10/group). Graph depicts tumor volumes measured with a vernier caliper over the course of the experiment. (**C**) Patient-derived HNSCC tumor masses (35 mg/site) were implanted subcutaneously on both flanks of athymic nude-*Foxn1*^*nu*^ mice. Ten mice per group were treated by oral gavage with GZ17-6.02 (30 mg/kg/d for first 7 days, and dose increased to 50 mg/kg/d to improve antitumoral effect) or vehicle control (saline) for 19 days. The graph depicts fractional tumor volumes over the course of the experiment. (**D**) Representative immunoblot of patient-derived xenograft lysates demonstrates decrease in p-ERK1/2 levels. Densitometric analysis of p-ERK1/2 relative to density of loading control (β-tubulin) of GZ17-6.02 treated tumors (n = 8) compared relative to vehicle control (n = 8) were graphed. Error bars represent ± SEM.

GZ17 formulations have not been tested in animal models. To determine the efficacy of GZ17 formulations *in vivo*, we treated subcutaneous OSC19 tumors in athymic nude mice with intratumoral injections (15 mg/kg/day) of GZ17-6.02 formulations. Among the formulations tested, GZ17-6.02 treatment was most efficacious with significant tumor volume reduction (p < 0.001, Fig. 6A). To assess GZ17-6.02 in an immunocompetent host, a syngeneic SCC model was employed. SCC vII/SF syngeneic tumors are highly aggressive requiring higher doses for testing. Thus, in this model, GZ17-6.02 was delivered by oral gavage, and a greater dose (100 mg/kg/day). Again, a significant decrease in tumor volume was demonstrated by GZ17-6.02 administered by oral gavage (p < 0.001, Fig. 6B). GZ17-6.02 receiving mice were active, curious and demonstrated normal, healthy behavior. There were no differences in mouse weights between treatment groups (Supplemental Figure 8), and no adverse effects from treatment were observed. This indicates GZ17-6.02 is well tolerated.

Finally, the response of GZ17-6.02 in patient-derived xenograft (PDX) tumors was evaluated. GZ17-6.02 was administered by oral gavage at a lower dose of 30 mg/Kg/d for 8 days. The dose was increased to 50 mg/kg/d for the remainder of the study to improve the antitumoral response. GZ17-6.02 demonstrated significant anti-tumor effects compared to the vehicle control treated mice (p < 0.01, Fig. 6C). In addition, tumors from GZ17-6.02 treated mice demonstrated a significant decrease in pERK1/2 levels (p = 0.0006, Fig. 6D). These data indicate substantial anti-tumor effects of GZ17-6.02 in HNSCC preclinical models.

Although promising anti-tumor effects are observed in preclinical models, a shortcoming of the current study is the lack of a clinical trial. Nevertheless, this study paves the way for future clinical studies.

## Discussion

The safety and proven efficacy of nutraceuticals has spurred research enthusiasm^36^. In HNSCC, as well as other tumor types, the usefulness of combination therapy has also gathered great attention as targeting multiple components of a signaling pathway more effectively mitigates tumor progression^37^. However, few studies have examined combining nutraceuticals to potentiate their effects^18^, and none have studied a nutraceutical combination for HNSCC. As such, we hypothesized a combination therapy of known anti-cancer nutraceuticals could potentiate antitumor effects. Nonetheless, overly complex combinations of a plethora of nutraceuticals diminishes, and may even worsen, expected effects^38^. In this work, the nutraceutical curcumin, which had demonstrated putative efficacy in previous combinatorial approaches, was combined with two nutraceuticals proposed to have anti-cancer activity, *Arum palaestinum* and *Peganum harmala*. Our results demonstrate the rationally designed combination of GZ17-6.02, a blend of curcumin, harmine, isovanillin, is superior in anti-cancer efficacy compared to any single agent, and demonstrated robust anti-tumor effects in preclinical models.

In HNSCC, curcumin enhances cisplatin efficacy by increasing caspase 3/9 activity^39^, and in breast cancer, curcumin decreases proliferation by inhibiting phosphorylation of ERK1/2^40^. In other tumor types, curcumin demonstrated promising combinatorial effects when combined with both natural compounds and effective drugs^18^. Our study corroborated these findings in that the combination therapy with GZ17-6.02 induced significant apoptosis through caspase 3, as well as inhibited phosphorylation of ERK1/2. Curcumin inhibits the production of reactive oxygen species in macrophages. This is an important step preventing macrophage-mediated inflammation-induced tumor progression^41^. Further, curcumin has been shown to induce the differentiation of macrophages to the tumor inhibitory M1 phenotype^42^. To this end, we demonstrated a significant reduction in macrophages in our MMD model. Harmine, the naturally occurring β-carboline, has anti-tumor and anti-angiogenic effects by intercalating and damaging DNA, and inducing tumor-suppressor p53^24,43,44^. Our studies substantiate these findings as GZ17-6.02 significantly decreased HNSCC proliferation and angiogenesis. Further, it reduced macrophage viability and infiltration into tumors *in vitro*. Isovanillin is a phenolic aldehyde, an isomer of vanillin that has not been extensively studied in the published literature for anticancer effects. However, isovanillin is known for its antioxidant effects^45^, and has been proposed as a potential antitumor agent^22^. In our preliminary studies, the combination of harmine and curcumin was potentiated by isovanillin, and, as such, we established the use of all three in the combined therapeutic.

As cancer pathogenesis is complex with many oncogenic pathways, the ability of an anti-cancer formulation to target multiple pathogenic steps is greatly desirable^37^. GZ17-6.02 decreases multiple signaling cascades, such as Src, ERK1/2, AKT, Stat-2, EGFR, and Chk-2. With molecular docking analyses, GZ17-6.02 components curcumin and harmine demonstrate high binding affinity with the active domains of EGFR, ERK1/2, and Akt1. Mutations in cancer related genes, including Akt1 and EGFR, and intratumoral heterogeneity can induce chemotherapy resistance^46,47^. The simultaneous binding of two or more ligands to the same target could be helpful in alleviating such resistance. Further, cisplatin resistance remains a significant hurdle for HNSCC treatment. We demonstrate the potentiated effects of standard of care cisplatin^35^, with GZ17-6.02. Previous studies indicate the improvement of cisplatin therapy with inhibition of Chk-2^48^, and we demonstrate the deactivation of Chk-2 with GZ17-6.02 administration, providing a possible explanation to the potentiation we observed.

Whereas nutraceuticals are often limited by bioavailability, we demonstrate the combination therapy GZ17-6.02 decreases growth of HNSCC xenograft tumors, syngeneic SCC tumors in an immunocompetent mouse model, and patient-derived xenografts of HNSCC. In establishing an *in vivo* dose, 15 mg/kg/day was initially tested by intratumoral injection. When the treatment was delivered via oral gavage, the dose was increased to 100 mg/kg/day in the syngeneic model. To assess a lower dose by oral gavage, 30 mg/kg/day was initially tested in the PDX model, however, to improve the antitumor efficacy, the dose was increased to 50 mg/kg/day on the eighth day. In all models, formulations were well tolerated in mice, mouse weight was consistent between treatment arms, and no adverse effects were observed. This tolerability is supported by our *in vitro* findings of a high ED_50_ in the non-cancerous cell line Het1A.

Therefore, the potentiated effects observed through combination *in vitro* align with the significant anti-tumoral responses in animal models. Further, GZ17-6.02 significantly decreases phospho-ERK1/2 levels *in vivo*, which supports the *in vitro* mechanistic experiments, and the molecular docking analyses. A limitation to our study is the lack of comparing GZ17-6.02 with single agents or combinations of two agents *in vivo*. However, these agents have had little clinical efficacy as single agents in patients, and no toxicities were observed in combination therapy in the *in vivo* models we assessed. Thus, we demonstrate GZ17-6.02 to be an effective anti-tumor therapy for the treatment of HNSCC in preclinical models, and pave the way for future clinical trials using this combination.

## Materials and Methods

### Cells and Reagents

Well characterized HNSCC cell lines were used in this study^49^. HNSCC lines HN5, UM-SCC-1 and OSC19, immortalized esophageal Het1A line from cancer free patient, glioblastoma line U87, and murine SCC/vII maintained in high glucose DMEM (Corning, NY, USA) with 10% heat-inactivated fetal-bovine serum (FBS). Lung cancer lines 201T and A549 grown in Basal Eagle Media (for 201T) and Hams F12K media (for A549) both supplemented with 10% FBS. All cells were grown for no more than 10-12 passages. HUVECs maintained in endothelial basal medium-2 supplemented with endothelial growth media-2 SingleQuot Kit (Lonza, Basel, Switzerland) per manufacturer’s instructions. Primary HNSCC tumors were collected under the auspices of the Biospecimen repository core at the University of Kansas Cancer Center with written informed consent from patients, using protocols approved by Institutional Review Boards at the University of Kansas Medical Center (KUMC), and all experiments were performed in accordance with the relevant guidelines and regulations.

The plant extract, GZ17-S, was provided by Genzada Pharmaceuticals (Sterling, KS) and prepared by combining *Peganum harmala* seeds, curcumin, and *Arum Palaestinum* extract, using a previously published protocol^22^. GZ17-5.00 (Afaya Plus) was provided by Genzada Pharmaceuticals, and prepared by combining nutraceutical components: harmine (Indofine, Hillsborough, NJ), piperonal, curcumin, hydroxymethyl furfual, limonene, benzyl nitrile, citraconic anhydride, isovanillin, and methylpyroglutamate (all from Sigma-Aldrich, St. Louis, MO). In the extraction procedure, iodine solution (LabChem Inc, Zelienople, PA), beta-sitosterol (MP Biomedicals, Santa Ana, CA), linolenic acid (TCI Chemicals, Portland, OR), and diallyldisulfide (Sigma-Aldrich) were used. GZ17-6.02 was prepared by combining curcumin, harmine and isovanillin (Sigma-Aldrich) (Supplemental Table 1).

Cisplatin was obtained from Fresenius Kabi (purchased by KUMC Pharmacy, Kansas City, KS). Tofacitinib was purchased from Selleckchem (Houston, TX). Recombinant human epidermal growth factor receptor was purchased from Fisher Scientific (Gibco, PHG0314, Waltham, MA).

### Cytotoxicity assay

Cells (4 × 10^3^ cells/well, 96-well plate) were treated in triplicate with GZ17-formulations or indicated components at various concentrations, and cell proliferation assessed using CyQUANT assay kit (Life Technologies, Waltham, MA) per manufacturer’s instructions. ED_50_ calculated using non-linear curve fit with GraphPad Prism software version 6.03 (GraphPad Software Inc., San Diego, CA).

HNSCC cells treated with cisplatin (4 µM), GZ17-6.02 (ED_50_ of respective cell type), combination cisplatin and GZ17-6.02, or vehicle control for 72 h. Cell viability assessed using CyQUANT.

To assess the efficacy of GZ17-06.02 to potentiate the effects of radiation on HNSCC, OSC19 (2 × 10^3^ cells/well in 96-well plates) were treated with 3, 6, and 9 Gy of radiation. Plates were exposed to gamma radiation (J.L. Shepherd and Associates Mark I Model 68 A cesium-137 source irradiator; dose rate = 2.9 Gy/min). Media was aspirated, and cells were treated with GZ17-06.02 ED50 concentration or vehicle control in DMEM with 10% FBS.

### Migration and invasion assay

Cells (2 × 10^4^ cells per insert) were seeded in trans-well inserts with 8 μm pores (Becton Dickinson, Franklin Lakes, NJ). For invasion assay, a layer of diluted Matrigel (2 mg/ml) in DMEM (BD Biosciences, San Jose, CA) was placed in the insert. Cells in serum-free media were seeded onto Matrigel layer for invasion or directly onto insert for migration assay. The inserts were placed in duplicate holding-wells containing GZ17-formulations (ED_50_) in complete media for 24 h. Treated cells were plated in parallel to assess viability using CyQuant. The number of cells that moved to other side of membrane were counted after fixation and staining with Hema3 kit (Fisher Scientific). The numbers of invading or migrating cells were normalized to cell viability. Additional details provided in supplemental methods.

### Tubule Formation Assay

HUVEC cells were plated on Matrigel in triplicate with GZ17-6.02 or vehicle control (15 × 10^3^ cells/well of 96-well plate). After 6 h, images were taken from 5 random fields per well. Images analyzed using Pipeline software version 1.4 (Medical College of Wisconsin, Milwaukee, WI) according to published instructions to quantify total tube length^50^.

### Macrophage infiltration and viability

To test the effect of GZ17-formulations in attenuation of macrophage infiltration into the tumor, we used the metastasic mimetic device (MMD) as previously described^33^. Briefly, 2.5 × 10^5^ OSC19 cells were embedded in rat tail collagen in the MMD and allowed to gel overnight. 5 × 10^5^ Thp1 cells were plated into the outer chamber of the device in serum containing medium. The collagen plug was imaged or processed for immunofluorescence analyses after 48 h. The number of macrophages that invaded the HNSCC containing collagen plugs were quantified using ImageJ software. Viability of Thp1 cells were assessed by counting cells on a hemocytometer with using trypan blue dye exclusion. HNSCC cell viability was assessed by imaging cells stained with 0.1 µM Calcein AM dye for 60 min at 37 °C in the dark.

### Human phospho-kinase array

Human phospho-kinase array (ARY003, Proteome Profiler™, R&D Systems, Minneapolis, MN) used to identify signaling molecules regulated by GZ17-formulations per manufacturer’s instructions. Membranes imaged by autoradiography, and ImageJ quantified signal intensity. Additional details provided in supplemental methods.

### Immunoblotting

Cells (3 × 10^5^ cells/60 mm dish) were treated with GZ17-formulations (ED_50_) for 72 h and lysed in RIPA buffer containing protease/phosphatase inhibitors (cOmplete, Mini, Roche, Indianapolis, IN). For inhibition of EGF stimulation, cells were serum starved for 48 h, and then treated with GZ17-6.02 (ED_50_) for 2 h. EGF (10 ng/mL) was applied for 5 m, and then cells were harvested in RIPA buffer containing protease/phosphatase inhibitors on ice Proteins separated by SDS-PAGE, transferred onto nitrocellulose membranes, and probed with p-ERK1/2 (p44/42-pMAPK; Thr202/Tyr204), total ERK1/2, cleaved PARP, caspase-3 (Cell Signaling, Danvers, MA) and β-tubulin (Fisher). Immunoreactivity detected using anti-mouse or anti-rabbit IgG conjugated to Dylight-680 or −800 (Fisher). Protein bands detected using Li-Cor Odyssey protein imaging system (Li-Cor Biotechnology, Lincoln, NE) and quantified using ImageJ. Additional details provided in supplemental methods.

### Computational Molecular Docking

Ligands designed using ACD/ChemSketch software (ACDlabs, Ontario, Canada). Crystal structures of proteins downloaded from RCSB-Protein Data Bank (http://www.rcsb.org/pdb/home/home.do). AutoDock-Vina employed for molecular docking using protein and ligand information. During the docking procedure, proteins were considered as rigid and ligands as flexible. The docking pose with lowest binding energy and highest binding affinity was aligned with the protein structure. Additional details provided in supplemental methods.

### Cell cycle analysis

OSC-19 cells (2 × 10^5^) treated in triplicate with ED_50_ concentrations of GZ17-formulations for 72 h. Cells were washed with PBS, fixed with 70% ethanol, stained with propidium iodide (0.02 mg/ml in PBS with 0.1% TritonX-100) (Life Technologies), and subjected to cell cycle analysis using Accuri C6 Flow Cytometer (BD Biosciences). Data were analyzed using the software provided by the manufacturer and samples analyzed in triplicate.

### *In vivo* studies

Animal care was in strict compliance with IACUC guidelines at the University of Kansas Medical Center, and all experimental protocols were approved by the IACUC. Mice were housed in individually ventilated cages in a sterile environment, and all experimental work was conducted during daytime hours. All outcome measures were assessed by a blinded observer. To assess *in vivo* antitumor efficacy of GZ17-formulations, 1 × 10^6^ HNSCC (OSC19) cells were injected subcutaneously into the flank of athymic nude-*Foxn1*^*nu*^ mice (Harlan Sprague Dawley, Indianapolis, IN). Tumor bearing mice were randomized once tumor volume reached 5.0 mm^3^. Mice treated with 15 mg/kg/day of GZ17-formulations or with saline control by 50 µL intratumoral administration once daily, five days/week. Tumor diameters measured in two perpendicular dimensions and volume calculated as previously described, briefly (tumor volume = long dimension x (short dimension)^2 × 0.52)^51^. Animals euthanized by CO_2_ asphyxiation followed by pneumothorax.

To assess GZ17-6.02 in immunocompetent host, a syngeneic HNSCC model was used^52^. 1 × 10^6^ SCC/vII cells were inoculated in the flank of C3H mice. Tumors were established for 6 days, and mice randomized into treatment groups (n = 10/group). Treatment was delivered suspended in 1% carboxymethylcellulose (CMC) by oral gavage (100 mg/kg/day). Mouse weight and tumor volumes were assessed three times per week.

To assess GZ17-6.02 in patient-derived HNSCC xenografts, 35 mg of primary HNSCC tissue was surgically implanted subcutaneously into flanks of athymic nude-*Foxn1*^*nu*^ mice under inhalant isofluorane anesthesia. Intraoperatively animals were administered analgesic Buprenorphine SR at 0.3 mg/kg and tofacitnib (to reduce natural killer cell counts) at 15 mg/kg via subcutaneous injections. Established tumors were passaged twice through mice before implantation for study. Tumor bearing mice were randomized in treatment groups (n = 10/group). Mice were treated with 30 mg/kg GZ17-6.02 or 1% carboxymethycellulose control via oral gavage once daily, five days/week. The dose was increased to 50 mg/kg/day on treatment day 8 to improve antitumoral response. As PDX models have variable growth rates, data presented for *in vivo* studies as fractional tumor volume. Tumors were excised, snap frozen in dry ice and analyzed by immunoblotting.

### Statistical Analysis

All results are cumulative from three independent experiments, unless otherwise indicated. Data analyzed using nonparametric Mann-Whitney tests. Analyses performed using Graphpad Prism Version 6.03.

### Data Availability

The data generated or analyzed during this study are available from the corresponding author on reasonable request.

## Electronic supplementary material

Supplementary Methods, Tables, and Figure Legends
