# Supplementary material for: Potent Antitumor Effects of a Combination of Three Nutraceutical Compounds
Source: Sci Rep. 2018 Aug 15;8:12163. doi: 10.1038/s41598-018-29683-1 (PMC6093880; doi:10.1038/s41598-018-29683-1)

Potent Antitumor Effects of a Combination of Three Nutraceutical Compounds

Vikalp Vishwakarma*, Jacob New*, Dhruv Kumar, Vusala Snyder, Levi Arnold, Emily Nissen, Qingting Hu, Nikki Cheng, David Miller, Ahia Rael Thomas, Yelizaveta Shnayder, Kiran Kakarala, Terance Ted Tsue, Douglas A Girod, Sufi Mary Thomas

Supplementary Information

Methods in Detail

Migration and invasion assay

HNSCC cells were seeded in trans-well inserts with a pore size of 8 μm (Becton Dickinson, Franklin Lakes, NJ), at a density of $2x10^4$ cells per insert suspended in serum-free media. For the invasion assay, Matrigel (BD Biosciences, San Jose, CA) was diluted with serum-free DMEM in a ratio of 1:5 to final concentration of 2 mg/mL, and 100 μL was pipetted into the trans-well insert and allowed to solidify at 37°C for 30 min. HNSCC cells, at a density of $2x10^4$ cells per insert suspended in serum-free media were seeded on top of the Matrigel layer for the invasion assay or directly onto the insert for the migration assay. The inserts were placed in duplicate holding-wells containing GZ17-06.02, -05.00 or –S at their ED_{50} concentrations, or vehicle control alone for 24h in complete media. Cells with treatments were plated in parallel in 96-well plates to assess cell viability using the CyQuant kit (Life Technologies). The number of cells that migrated through matrigel was counted in 4 fields of each well at 100X magnification after hematoxyllin and eosin staining using the Hema3 kit (Fisher Scientific, Waltham, MA). The number of cells that migrated through the insert was normalized to the cell viability. The fold change in HNSCC migration and invasion in treatment arms relative to the vehicle control was determined and results graphed of three independent experiments.

Human phospho-kinase profiler array

In order to identify signaling molecules regulated by GZ17-formulations, we used a Human phospho-kinase array kit (ARY003, Proteome Profiler™, R&D Systems, Minneapolis, MN) according to the manufacturer's instructions. Briefly, OSC19 ($2 x 10^5$)

cells were treated with GZ17-06.02, -05.00, -S or vehicle control for 48h. Cells were rinsed three times with chilled PBS and lysed using supplied lysis buffer and protein was quantitated using a standard Bradford absorbance assay (Sigma-Aldrich). Array buffer containing 300μg of protein in 1 ml was applied to membranes previously treated with a blocking reagent. Streptavidin-HRP conjugate treated membranes were subjected to autoradiography after signals were developed with enhanced chemiluminescence reagent. ImageJ 1.46r software (NIH, Bethesda, MA) was used to quantify the intensity of the signals.

Immunoblotting

OSC19 cells ($3x10^5$) were seeded in 100 mm tissue culture dishes. Cells were treated with ED_{50} concentrations of GZ17-06.02, -05.00, -S or vehicle control for 72 h. Cells were washed three times with PBS and lysed in RIPA lysis buffer (10 mmol/L Tris-HCl (pH 7.6), 50 mmol/L NaCl, 5 mmol/L EDTA, 50 mmol/L NaF, 30 mmol/L sodium pyrophosphate, 1 mmol/L sodium vanadate) and phospho-protease minitab (Roche, Indianapolis, IN). Cells were sonicated and clarified at 13,000 rpm at 4°C for 15 min. The protein concentration was determined using Bradford reagent. Proteins were separated by SDS-PAGE, transferred onto nitrocellulose membranes, and probed with p-ERK1/2 (P44/42-pMAPK; Thr202/Tyr204), cleaved PARP, caspase-3 (Cell Signaling, Danvers, MA) and β-tubulin (Thermo Scientific, Waltham, MA). Immunoreactivity was detected using anti-mouse or anti-rabbit IgG conjugated to Dylight 680 or 800 fluorochromes (Thermo Scientific,), respectively. Blots were visualized on Odyssey imager (LiCor, Lincoln, NE). Quantitation of immunoreactive signals was done by densitometry using

ImageJ 1.46r software (NIH). The ratio of protein expression to β-tubulin in each lane was calculated and presented relative to the respective controls within each experiment.

Computational Molecular Docking

Ligands (curcumin and harmine) were designed using ACD/ChemSketch software (ACDlabs, Ontario, Canada). Open Babel software (Open Babel 2.3.1, Open Babel: The Open Source Chemistry Toolbox) was used to convert 3D structure of ligand (.MOL file) into .PDB file. Ligands were optimized using Graphical User Interface program AutoDock Tools4 (Scripps Research Institute, La Jolla, CA). Crystal structures of proteins were downloaded from RCSB-Protein Data Bank (http://www.rcsb.org/pdb/home/home.do) and the PDB files were converted to .txt files. Proteins were optimized using AutoDock Tools4.2. Intermediary steps, such as .pdbqt files preparation for protein and ligands, and grid box creation were completed using AutoDock Tools4.2 (Scripps Research Institute, La Jolla, CA). In addition, the program was used to assign charges, solvation parameters and fragmental volumes to the protein. AutoDock-Vina was employed for molecular docking using protein and ligand information along with grid box properties in the configuration file. During the docking procedure, proteins were considered as rigid and ligands as flexible molecules. The analysis of docking pose with lowest binding energy and highest binding affinity were extracted and aligned with receptor structure using AutoDock Tools4.2.

Combinatorial effect of GZ17-06.02 with radiation on HNSCC

To assess the efficacy of GZ17-06.02 to potentiate the effects of radiation on HNSCC, OSC19 ($2x10^3$ cells/well in 96-well plates) were treated with 3, 6, and 9 Gy of

radiation. Plates were exposed to gamma radiation (J.L. Shepherd and Associates Mark I Model 68A cesium-137 source irradiator; dose rate = 2.9 Gy/min). Media was aspirated, and cells were treated with GZ17-06.02 ED_{50} concentration or vehicle control in DMEM with 10% FBS. Cells were grown for 72 hours, then the media was removed and the plate frozen at -80°C. Assessment of cell proliferation across treatment groups in triplicate was performed using CyQUANT assay kit per the manufacturer's instructions. Fold change in cell survival relative to vehicle control was assessed from three independent experimental repeats.

Supplemental Tables and Figures

Supplementary Table 1

Supplemental Table 1: Synthetic components in GZ17-5.0 and GZ17-6.02

**GZ17-5.0**	
Component	*µg/mL of water*
Isovanillin	5.882
Beta-sitosterol	11.765
Linolenic Acid	4.059
Harmine	9.941
Hydroxymethyl furfural	5.529
Piperonal	2.000
Citraconic anhydride	2.235
Methylpyroglutamate	2.529
Curcumin	7.529
Benzyl nitrile	4.412
Limonene	2.941
**GZ17-6.02**	
Component	*mg/mL of ethanol*
Isovanillin	771
Harmine	130.3
Curcumin	98.7

Supplemental Table 2: Binding energy of GZ17-6.02 active components with molecular targets of HNSCC

	**EGFR**	**ERK1**	**ERK2**	**AKT-1**
Curcumin	-6.5	-8.4	-7.1	-6.6
Curcumin Glucuronide	-9.2	-11.3	-11.3	-10.7
Curcumin Sulfate	-7.2	-7.6	-7.9	-6.8
Harmine	-6.8	-7.0	-8.1	-6.3

Supplemental Figure 1

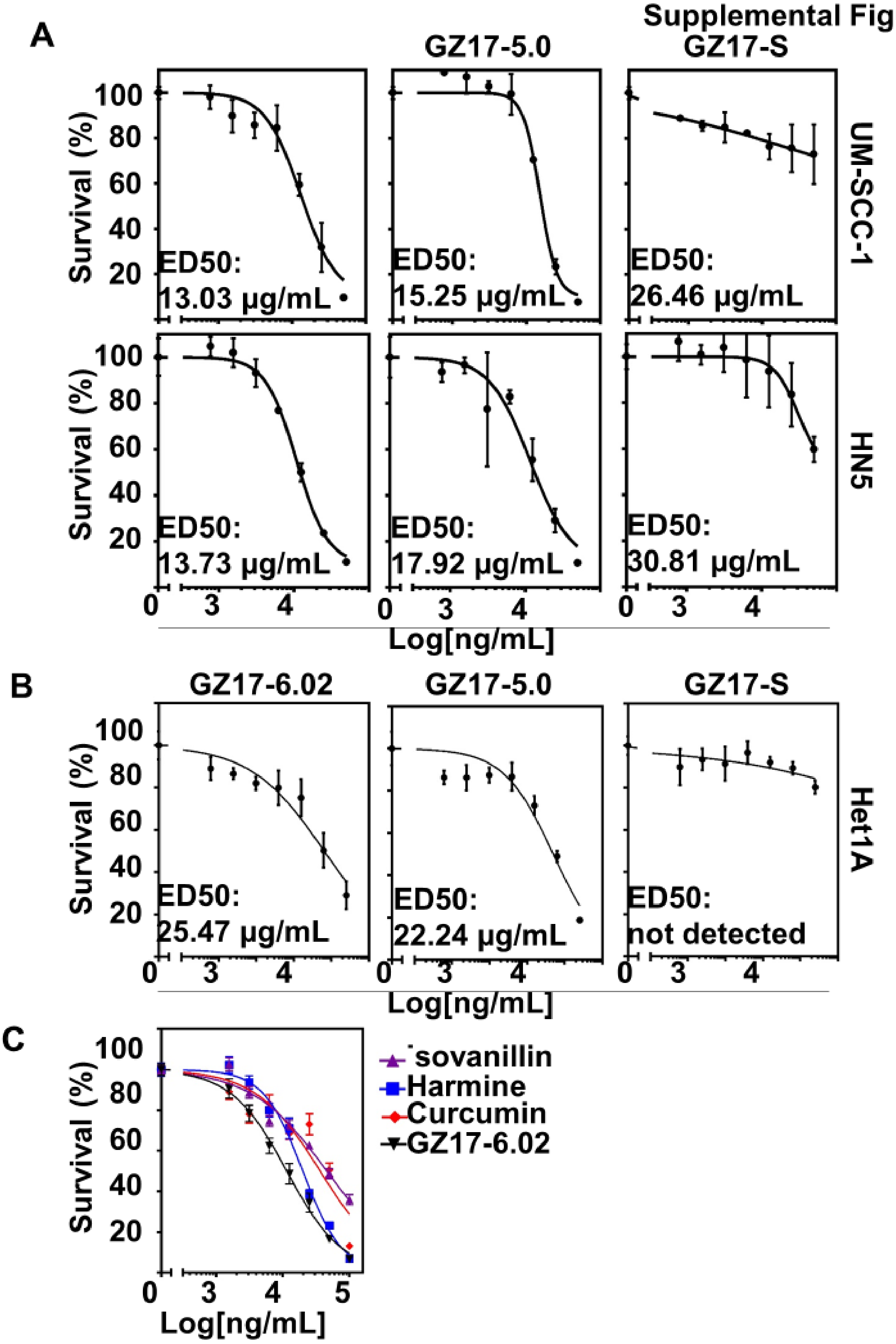

Supplemental Figure 1. GZ17 formulations potently inhibit proliferation of multiple HNSCC cell lines.

(A) HNSCC cells (UM-SCC1, HN5, 4×10^3 cells) and (B) cells from cancer-free patient (Het1A) were treated with various concentrations of GZ17-06.02, -05.00 and –S for 72 hours. Effective dose 50 (ED_{50}) was calculated with non-linear curve fit using GraphPad Prism software. Cumulative data represents three individual experimental repeats and error bars represent ± SEM.

(C) Bliss analysis of each individual component (Isovanillin, Harmine, or Curcumin) and combination of all three components. OSC19 (4×10^3 cells/well) were plated in triplicate and treated with various doses of GZ17-6.02 or individual component at proportional concentrations (0, 1.56, 3.13, 6.25, 12.5, 25, 50, and 100 ug/mL). This analysis indicates the non-linear curve fit for GZ17-6.02 is not the summed results of each individual components non-linear curve, which indicates GZ17-6.02 is additive but not synergistic.

Supplemental Figure 2

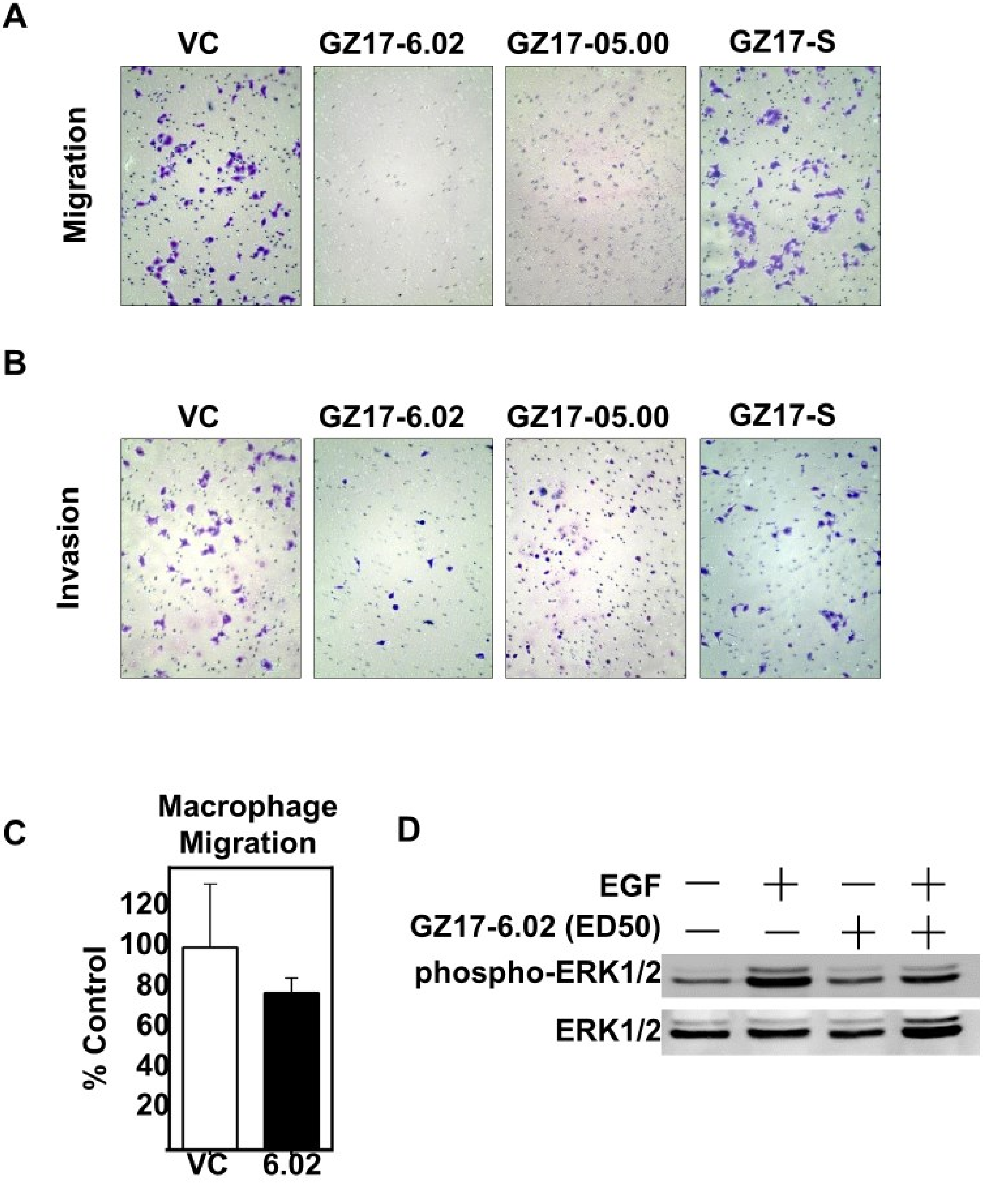

Supplemental Figure 2. GZ17-6.02 reduces migration, invasion, macrophage infiltration, and phosphorylation of ERK1/2.

(A) representative images of OSC19 migration transwell inserts with indicated GZ17 formulations. Images taken at 100X magnification of light microscope; OSC19 cells adherent to well were stained with Hema3 staining kit for contrast.

(B) Representative images of OSC19 invasion transwell inserts with indicated GZ17 formulations. Images taken at 100X magnification of light microscope; OSC19 cells adherent to well were stained with Hema3 staining kit for contrast.

(C) Thp1 cells (5×10^5) were plated into the outer chamber of the MMD and treated with GZ17-6.02 (60 ug/mL). The collagen plug was imaged, and the number of macrophages that invaded the HNSCC containing collagen plugs were quantified using ImageJ software. Cumulative data represents five individual experimental repeats and error bars represent ± SEM.

(D) Representative immunoblot of phospho-ERK1/2 and total ERK1/2 from OSC19 cells. These lysates were collected from OSC19 that were serum starved for 48 h, and then treated with GZ17-6.02 (ED50) for 2 h. EGF (10 ng/mL) was applied for 5 m, and lysates were harvested for immunoblot analyses.

Supplemental Figure 3

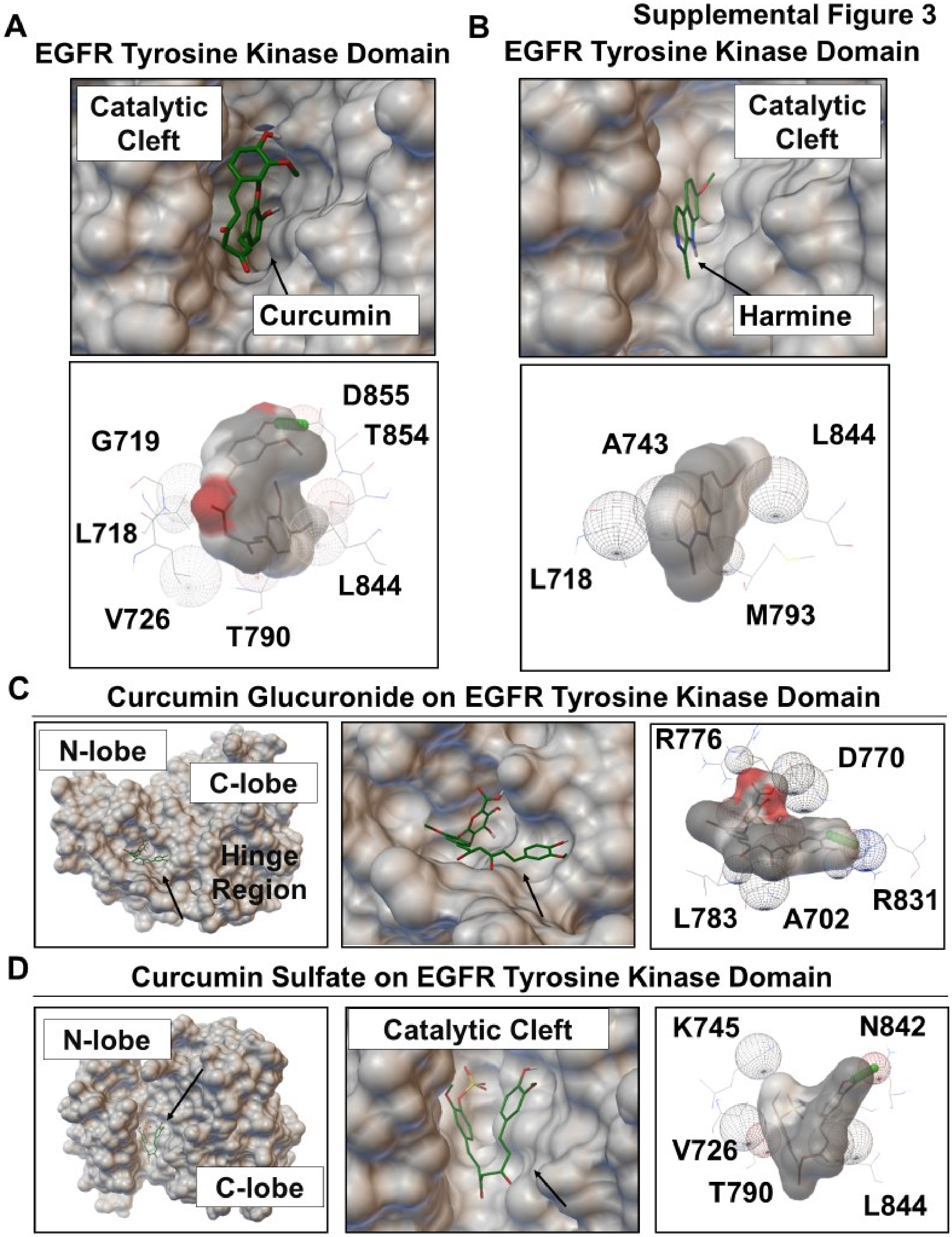

Supplemental Figure 3. GZ17-6.02 components and metabolites interact in active sites of EGFR tyrosine kinase domain.

(A) Curcumin displays best binding affinity in the catalytic cleft of EGFR tyrosine kinase domain, and interacts with described amino acid residues.

(B) Harmine demonstrates best binding affinity of different residues of the catalytic cleft of EGFR tyrosine kinase domain.

(C) Curcumin metabolite, curcumin glucuronide, exhibits best binding affinity for the EGFR kinase domain hinge region.

(D) Curcumin metabolite, curcumin sulfate, exhibits best binding affinity for the catalytic cleft of the EGFR tyrosine kinase domain.

Supplemental Figure 4

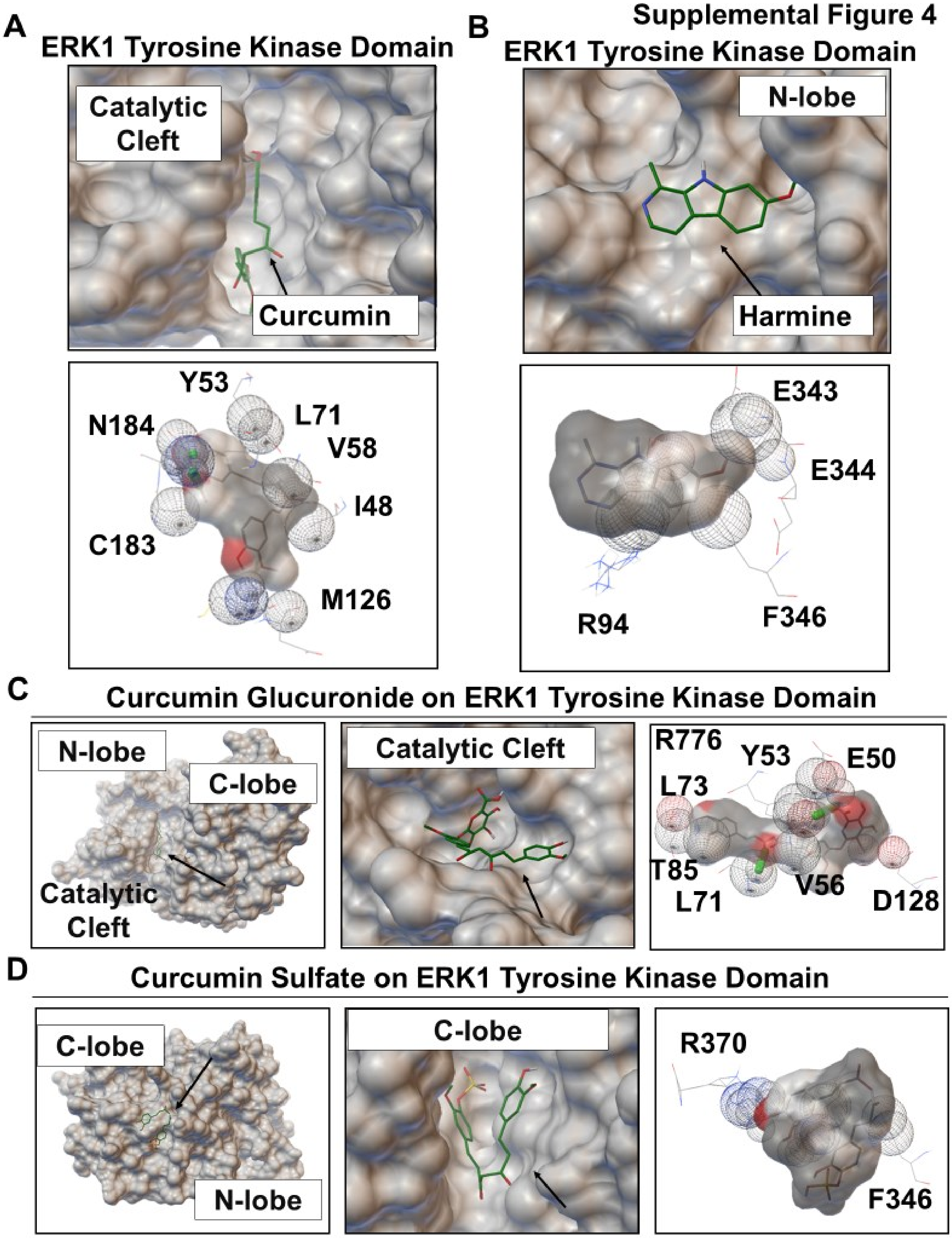

Supplemental Figure 4. GZ17-6.02 components and metabolites interact in active sites of ERK1 tyrosine kinase domain.

(A) Curcumin displays best binding affinity in the catalytic cleft of ERK1 tyrosine kinase domain, and interacts with described amino acid residues.

(B) Harmine demonstrates best binding affinity of different residues of the catalytic cleft of ERK1 tyrosine kinase domain.

(C) Curcumin metabolite, curcumin glucuronide, exhibits best binding affinity for the ERK1 catalytic cleft.

(D) Curcumin metabolite, curcumin sulfate, exhibits best binding affinity for the catalytic cleft of the ERK1 tyrosine kinase domain c-lobe.

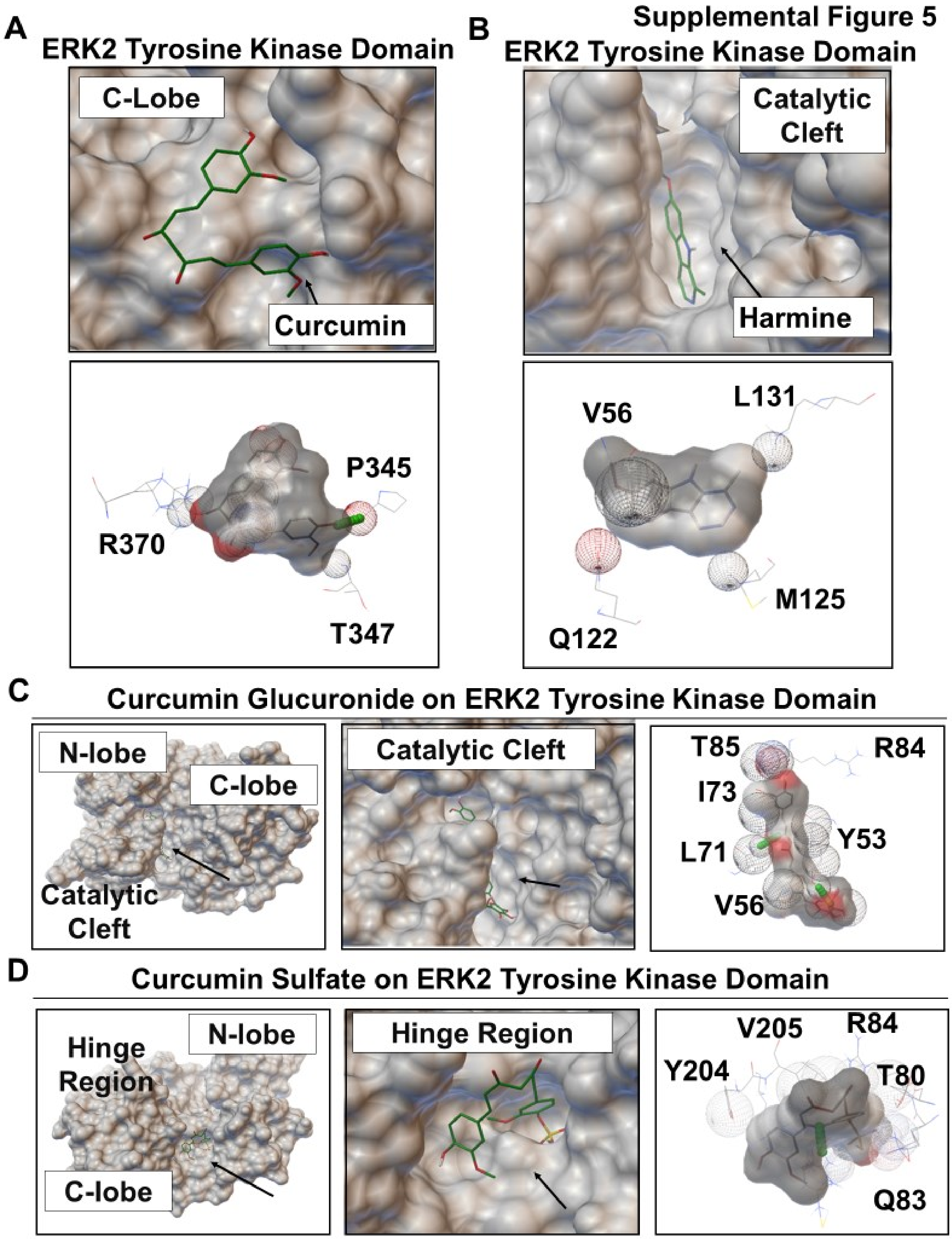
Supplemental Figure 5
A
ERK2 Tyrosine Kinase Domain
C-Lobe
Curcumin
P345
R370
T347
B
ERK2 Tyrosine Kinase Domain
Catalytic Cleft
Harmine
V56
L131
M125
Q122
C
Curcumin Glucuronide on ERK2 Tyrosine Kinase Domain
N-lobe
C-lobe
Catalytic Cleft
Catalytic Cleft
T85
R84
I73
Y53
L71
V56
D
Curcumin Sulfate on ERK2 Tyrosine Kinase Domain
Hinge Region
N-lobe
C-lobe
Hinge Region
V205
R84
Y204
T80
Q83

Supplemental Figure 5. GZ17-6.02 components and metabolites interact in active sites of ERK1 tyrosine kinase domain.

(A) Curcumin displays best binding affinity in the c-lobe of ERK2 tyrosine kinase domain, and interacts with described amino acid residues.

(B) Harmine demonstrates best binding affinity within the catalytic cleft of ERK2 tyrosine kinase domain.

(C) Curcumin metabolite, curcumin glucuronide, exhibits best binding affinity for the ERK2 catalytic cleft.

(D) Curcumin metabolite, curcumin sulfate, exhibits best binding affinity for the hinge region of the ERK2 tyrosine kinase domain.

Supplemental Figure 6

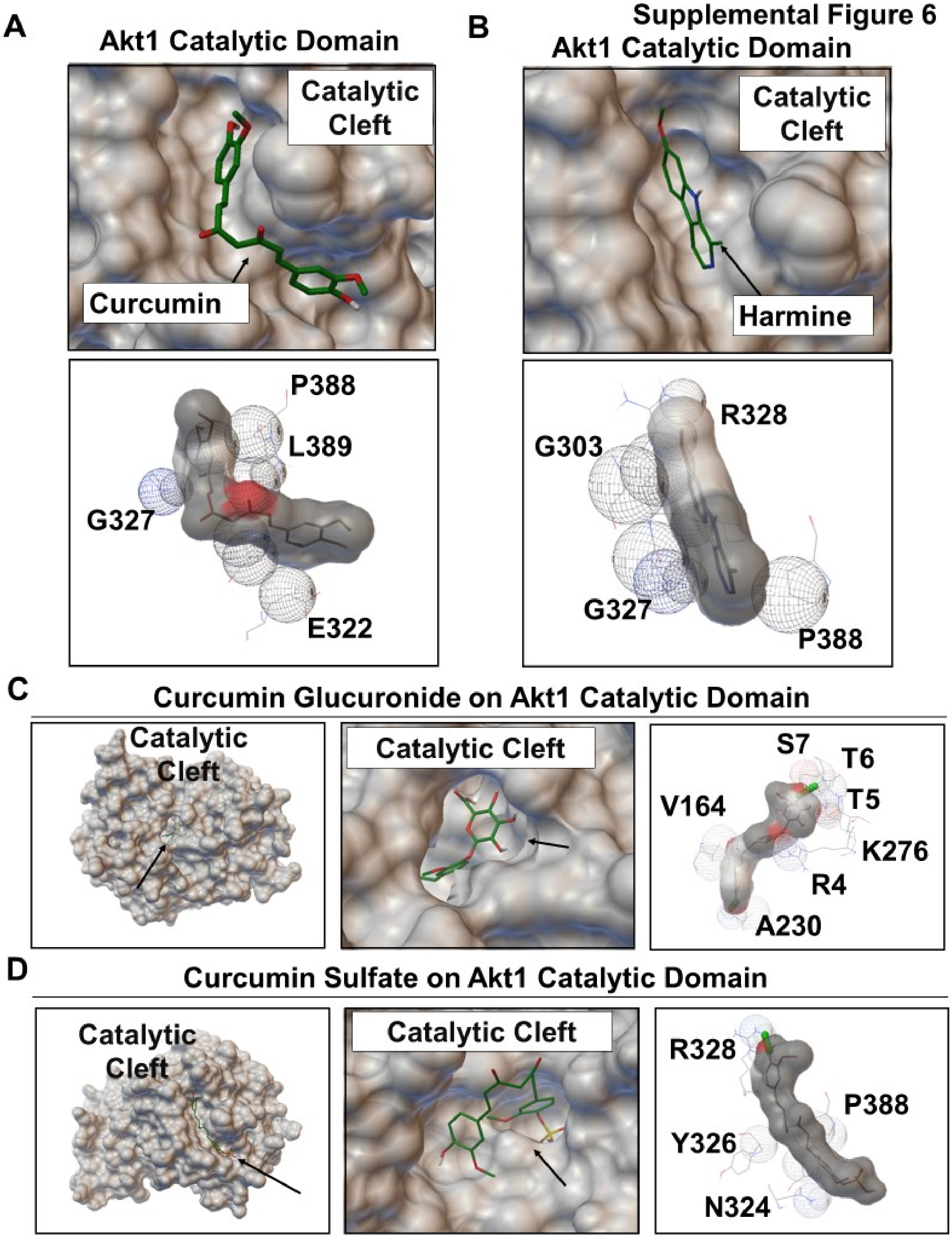

Supplemental Figure 6. GZ17-6.02 components and metabolites interact in active sites of Akt1 tyrosine kinase domain.

(A) Curcumin displays best binding affinity in the c-lobe of Akt1 catalytic domain, and interacts with described amino acid residues.

(B) harmine demonstrates best binding affinity within the catalytic cleft of Akt1 tyrosine kinase domain.

(C) Curcumin metabolite, curcumin glucuronide, exhibits best binding affinity for the Akt1 catalytic cleft.

(D) Curcumin metabolite, curcumin sulfate, exhibits best binding affinity for the catalytic cleft of the Akt1 tyrosine kinase domain.

Supplemental Figure 7

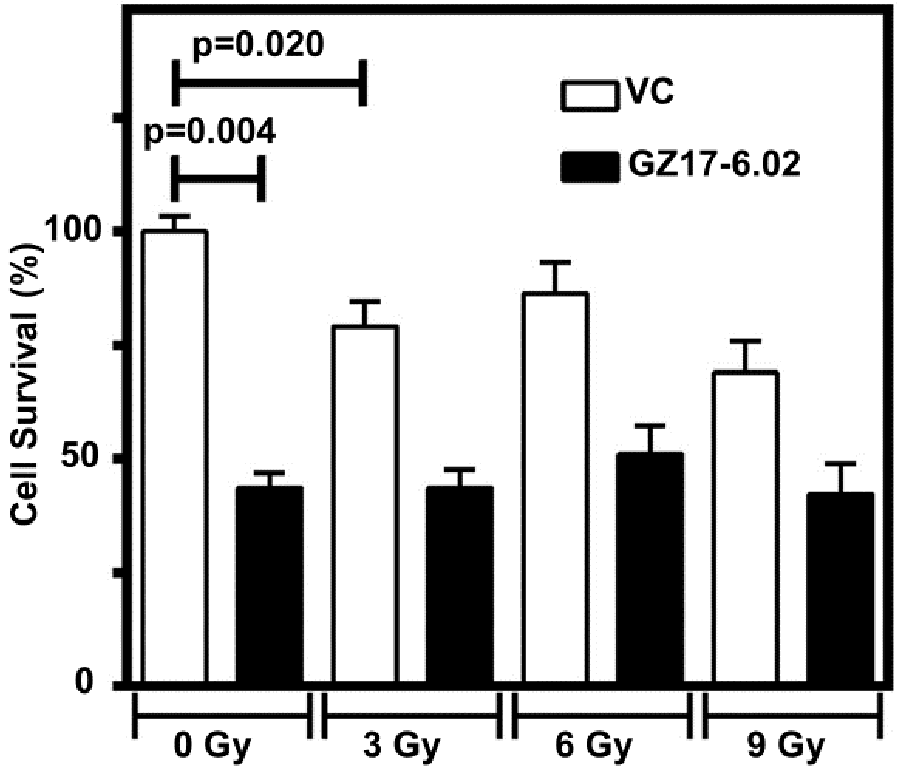

Supplemental Figure 7. GZ17-06.02 is more cytotoxic than 3, 6, or 9 Gy radiation. OSC19 cells were treated with single dose of 3, 6, or 9 Gy of radiation, and subsequently treated with GZ17-06.02 at ED_{50} concentration. Cell proliferation assessed at 72 hours. Graph represents proliferation relative to no irradiation control of 3 separate experimental repeats, plated in triplicate. Error bars represent ± SEM.

Supplemental Figure 8

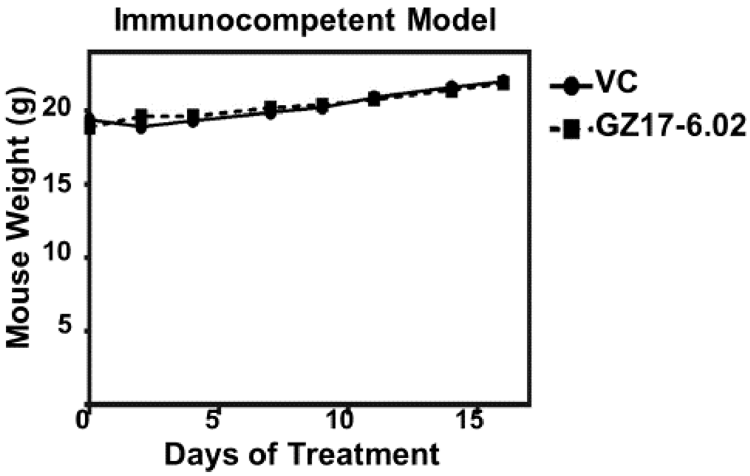

Supplemental Figure 8. GZ17-06.02 does not affect immunocompetent mouse weight

Overall mouse weight did not change between GZ17 treated (dashed line), and vehicle control (solid line) treated group throughout the course of the experiment. Error bars represent ± SEM.

Full-Length Blots

From Figure 1E

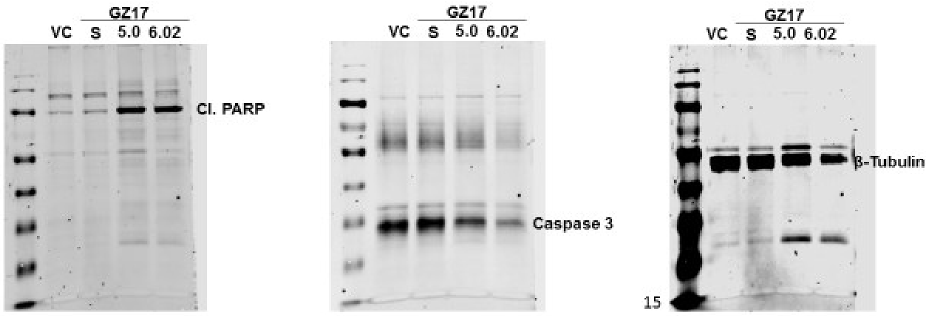

From Figure 3C

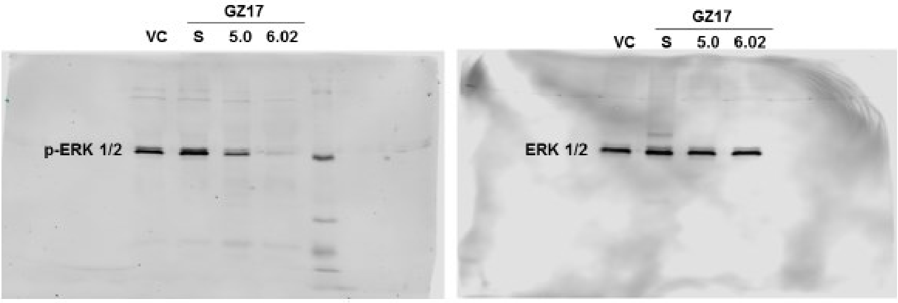

From Figure 6D

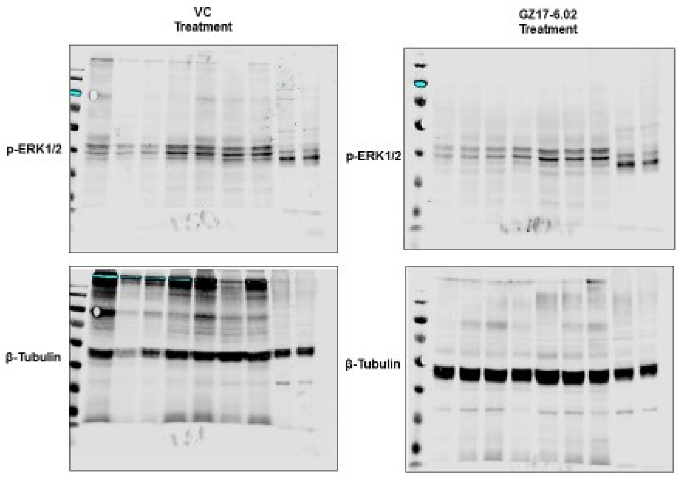

From Supplemental Figure 2D

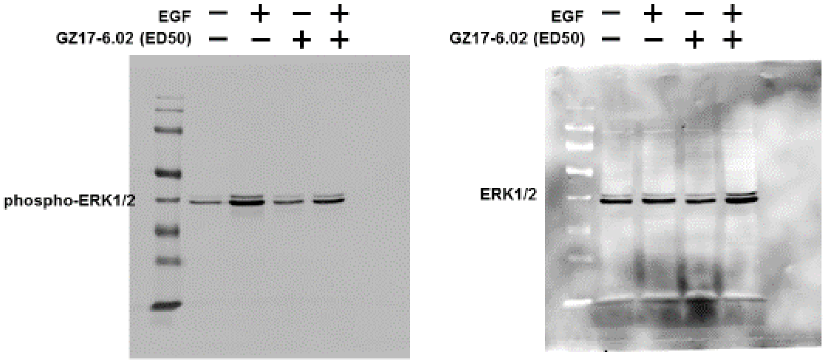

Supplement: Supplementary file 1 — Supplementary Methods, Tables, and Figure Legends [file 41598_2018_29683_MOESM1_ESM.pdf]
